# Morphometric and stereological methods for quantifying the coarse structural parameters of the ruminal tissues in sheep

**DOI:** 10.1080/23144599.2020.1807817

**Published:** 2020-09-08

**Authors:** Andrew Makanya, Ann Nancy Mills-Thompson, James Nguhiu-Mwangi, Jemimah Oduma, Rodi Ojoo

**Affiliations:** a Department of Veterinary Anatomy and Physiology, University of Nairobi, Nairobi, Kenya; b Department of Zoology, University of Johannesburg, Johannesburg, South Africa; c School of Veterinary Medicine, University of Ghana, Legon, Ghana; d Department of Clinical Studies, University of Nairobi, Nairobi, Kenya

**Keywords:** Sheep, rumen, morphometry, stereology, volumes, surfaces

## Abstract

In ruminants, the rumen is the largest and most significant fore-stomach. Stereological analysis of important structural parameters that may be used to assess the functional capacity of the rumen is lacking. In the current investigation, five rams were used to demonstrate the methods for quantifying salient structural parameters related to rumen function. The sheep were euthanized with 20% sodium pentobarbital intravenously, the rumen was dissected out and divided into the various sacs for gross examination, and fixation by total immersion in 10% formalin. Macroscopic ruminal surface area was estimated using the point-associated area method. Volumes of the ruminal tissues were estimated by the volume displacement method, while volume densities of the components of the ruminal wall were estimated by point counting methods. Tissue blocks for histology were obtained by systematic random sampling and processed to obtain vertical sections for surface area and volume estimations. Papillary densities and numbers were estimated from horizontal sections. The volume of ruminal tissue was 536.54 ± 80.52 cm^3^, the macroscopic surface area was 1091 ± 115.75 cm^2^ with a papillary packing density of 84.64 ± 10.99 cm^−2^. Average absolute surface area was 4726.74 ± 628.56 cm^2^. The total number of ruminal papillae per rumen was 92,884.91 ± 6216.46. The methods documented here provide the possibility of doing a detailed stereological analysis of ruminal tissue in different experimental or even pathological conditions.

## 1. Introduction

The rumen, which is the largest of the fore-stomach compartments and vital for microbial fermentation of forages and other feeds, as well as for production and absorption of volatile fatty acids, synthesizes 70–80% of the ruminant’s energy requirements [1]. The sheep rumen is a large and sacculated structure, which is divided by a series of pillars into five intercommunicating discrete ruminal sacs, namely the cranial, dorsal, ventral, caudodorsal blind and caudoventral blind sacs [2]. Histologically the ruminal wall is made up of an external tunica serosa, followed by a tunica muscularis, tunica submucosa and tunica mucosa. The tunica mucosa is studded with papillae, which have varying characteristics in the different ruminal sacs [3] and play a major role in increasing the absorptive surface area for enhanced ruminal function [4,5]. Previous histomorphological studies of sheep rumen have focused mainly on the qualitative dimensions with scanty quantitative data. Available morphometric investigations describe only localized portions rather than the entire rumen [3,6,7]. Therefore, information on the quantitative estimates of tissue parameters in the entire rumen is lacking. Design-based stereological techniques allow analysis of biological tissues in an unbiased way, with the result that the data obtained are free of systematic error [8,9].

This far, the morphometric estimates reported on the rumen are model-based and include quantities such as papillary length and thickness, among others. Currently, there are no elaborate methods for quantifying ruminal functional tissues, hence the erratic reports on rumen morphometry. In the current study, basic stereological principles were used to design unbiased methods for quantifying functional ruminal tissues. The techniques were combined with the simple, easy-to-use software; the STEPanizer® developed by Tschanz et al. [9]. However, only preliminary data are presented in this study to demonstrate the applicability of the new methods.

## 2. Materials and methods

### 2.1. Experimental animals

The treatment of animals and experimental set up of the present study were approved by the Biosafety, Animal Use and Ethics Committee of the Faculty of Veterinary Medicine, University of Nairobi, Kenya (approval certificate number FVM BAUEC/2014/025).

Five healthy castrated male dorper sheep aged between 12 and 15 months were selected from a larger herd raised for impaction experiments. The sheep were obtained from Gicheha Farm, Nairobi, Kenya. They were ear-tagged with individual identification codes and housed together in a stall within the Animal Unit of the Department of Clinical Studies, University of Nairobi for a 5-week acclimatization period. The sheep were fed with Rhodes grass and commercially produced concentrates (UNGA AFYA Meal®, UNGA Farm Care Ltd, and Nairobi, Kenya), supplemented with mineral lick and provided with water *ad libitum*.

### 2.2. Measurement of body weights

Measurements of body mass of sheep were done using a 50 kg capacity Salter® spring hanging scale. Each sheep was weighed while supported by a sling hooked to the weighing scale suspended to hang from an overhead metal bar.

### 2.3. Harvesting of tissues for macroscopic, histological and stereological analysis

After the acclimatization period each sheep was humanely euthanized with 20% sodium pentobarbital intravenously. The abdomen was immediately opened through a penetrating full-length ventral midline incision and the pelvis was opened by cutting through the pubis to expose the terminal part of the alimentary tract. The gastrointestinal tract including the fore-stomachs was removed from the abdominal cavity within a short time of opening the abdomen by severing the oesophagus several centimetres cranial to the diaphragm and the terminal rectal part.

**Figure 1.**
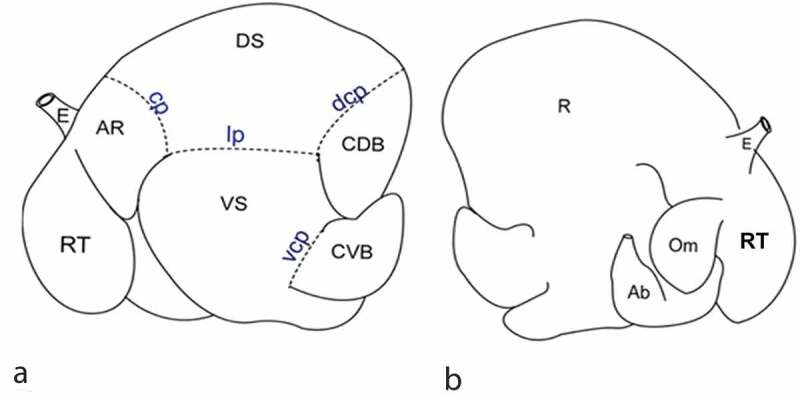
A and B: Schema of the ruminant stomachs showing the 5 ruminal sacs (a) as well as the abomasum (b). (a): Left side of the fore stomachs showing the ruminal sacs clearly demarcated from each other with dotted lines along their respective ruminal pillars. The ruminal sacs are atrium ruminis or cranial sac (AR), dorsal sac (DS), ventral sac (VS), caudodorsal blind sac (CDB) and caudoventral blind sac (CVB). The reticulum (RT) and the oesophagus (E) are also shown. The ruminal pillars indicated are the cranial pillar (cp), lateral longitudinal pillar (lp), dorsal coronary pillar (dcp) and the ventral coronary pillar (vcp). (b): On the medial aspect, the oesophagus (E), the rumen (R), the omasum (Om), the reticulum (RT) and the abomasum (Ab) are shown

The combined forestomachs were placed on a dissecting table with the parietal side facing up. An incision was made into the dorsal sac of the rumen beginning from the caudal end of the cranial groove just dorsal to the left longitudinal groove and continued to the cranial end of the caudal groove (Figure 1). With all parts of the fore-stomachs still attached to each other, a cut was made along the mesenteric border of the reticulum, omasum and abomasum to empty their contents. The stomachs were washed in clean water, and rinsed in phosphate buffered saline. The rumen was separated from the rest of the fore-stomach compartments by cutting through the rumeno-reticular junction and the rumeno-omasal junction, weighed and then photographed with mucosal surface facing upwards. The rumen was dissected into its five different sacs namely, the cranial sac or atrium ruminis (AR), dorsal sac (DS), ventral sac (VS), caudodorsal blind sac (CDB) and caudoventral blind sac (CVB) using the procedure described by McGavin and Morrill [10] with modifications (Figure 1). The cranial sac (AR) was separated from the dorsal sac by cutting lateral to the cranial coronary pillar. The CDB and CVB were dissected by cutting around the periphery cranial to the dorsal and ventral coronary pillars leaving the caudal pillar attached to the ventral sac. The VS was separated from the DS by cutting just dorsal to the right longitudinal and cranial pillars. The VS was identified with the right and left longitudinal pillars as well as cranial and caudal pillars, which guided correct anatomic orientation.

### 2.4. Fixation of tissues

The rumen sacs were fixed by total immersion in 10% neutral buffered formalin (NBF) fixative in individually labelled containers for ease of identification. The specimens were left in fixative for at least four days before further processing.

### 2.5. Measurement of reference volume of ruminal sacs

**Figure 2.**
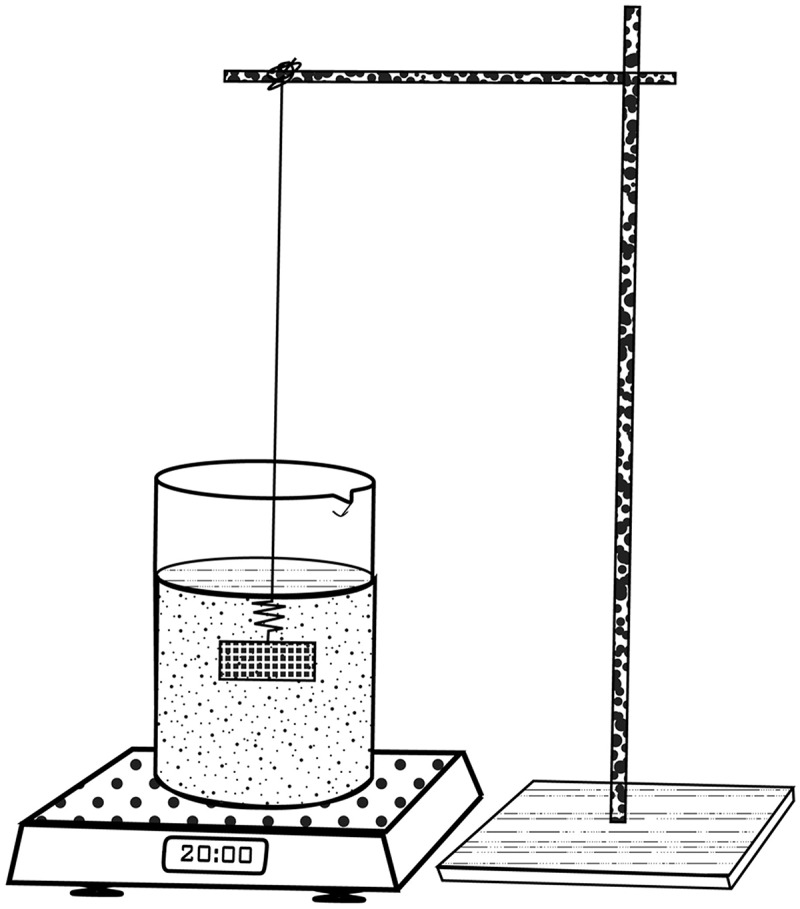
Schema demonstrating the Scherle method of volume estimation. Using a set of clamps, the ruminal sac is suspended with a thin wire and then submerged into a beaker containing 10% neutral buffered formalin. The beaker is placed on a weighing balance and the reading zeroed prior to the immersion of the ruminal sac. The reading is taken directly from the weighing balance and is equal to the volume of the ruminal sac in cm^3^

The reference volume of each ruminal sac was determined using the volume displacement method (Figure 2) described by Scherle [11]. In this method, a 500 ml Pyrex beaker was partially filled with 10% neutral buffered formalin and placed on a digital weighing scale (Mettler® PM4600 DeltaRange, Switzerland). The flap of ruminal tissue was hooked onto a thin metal wire clamped to a laboratory stand and submerged in the fluid. The weight recorded in grams on the electronic weighing balance corresponded to the reference volume in millilitres or cubic centimetres of the specific ruminal sac.

### 2.6. Measurement of the macroscopic surface area of ruminal sacs

The area of each ruminal sac at the gross level referred to as macroscopic surface area was then estimated using the point-associated area method [8]. Briefly, to estimate the area of each ruminal sac, a transparent counting grid with test points printed on it, which had a value representative of the area associated with a test point (a/p), was randomly yet completely superimposed on the mucosal surface of each ruminal sac. The number of test points hitting the surface of the ruminal sac was counted. The total area of each ruminal sac at the macroscopic level was then estimated by multiplying the total number of test points counted on each ruminal sac by the area associated with the test point on the counting grid. The total surface area of the entire rumen for each animal was obtained by adding the estimated values of each ruminal sac.

Thus the macroscopic surface area for each ruminal sac was obtained as,

$$S_{m}=\sum P^{*}a\left(p\right),$$

Where,

$S_{m}$ is the macroscopic surface area of the ruminal sac;

∑P is the total number of test points counted;

$a\left(p\right)$ is the area associated with a test point.

Alternatively, photographs of the ruminal sacs were taken with a ruler placed next to the sac. Subsequently, the lattice grid was placed on the photograph and the number of points counted. The area was then estimated as,

$${\text{S}}_{\text{m}}\text{=}\sum {\text{P}}^{\text{*}}\text{a}{\left(\text{p}\right)}^{\text{*}}1/{\text{M}}^{2}$$

where M is the magnification.

### 2.7. Sampling of the ruminal sacs for tissue processing and histological evaluation

Each ruminal sac, including the atrium ruminis or cranial sac (AR), dorsal sac (DS), ventral sac (VS), caudodorsal blind sac (CDB) and caudoventral blind sac (CVB) was sampled in a systematic uniform random manner [12].

**Figure 3.**
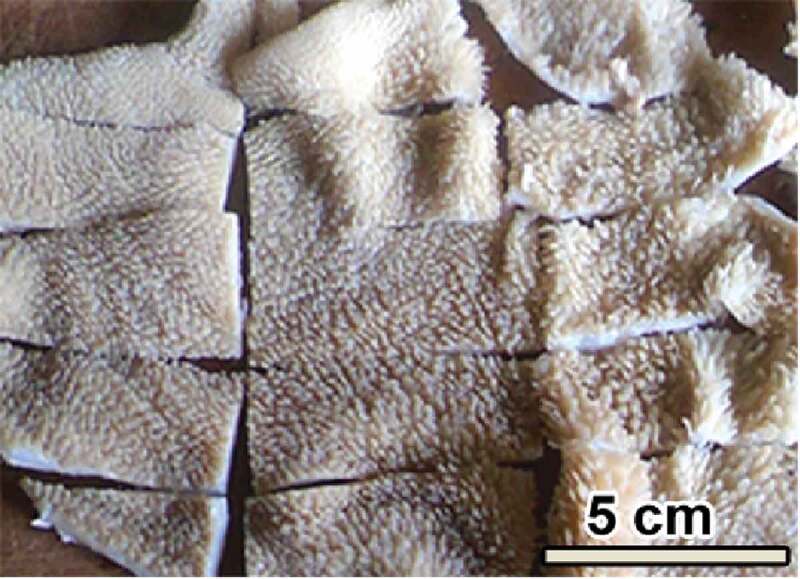
A ruminal sac cut into several slices of approximately equal sizes from which systematic random sampling was done. From the top left hand corner, the slices are numbered in an ascending manner starting from 1 (see Figure 4 below). The sampling interval was set at 5 and the first slice was picked at random in the interval 1–5

**Figure 4.**
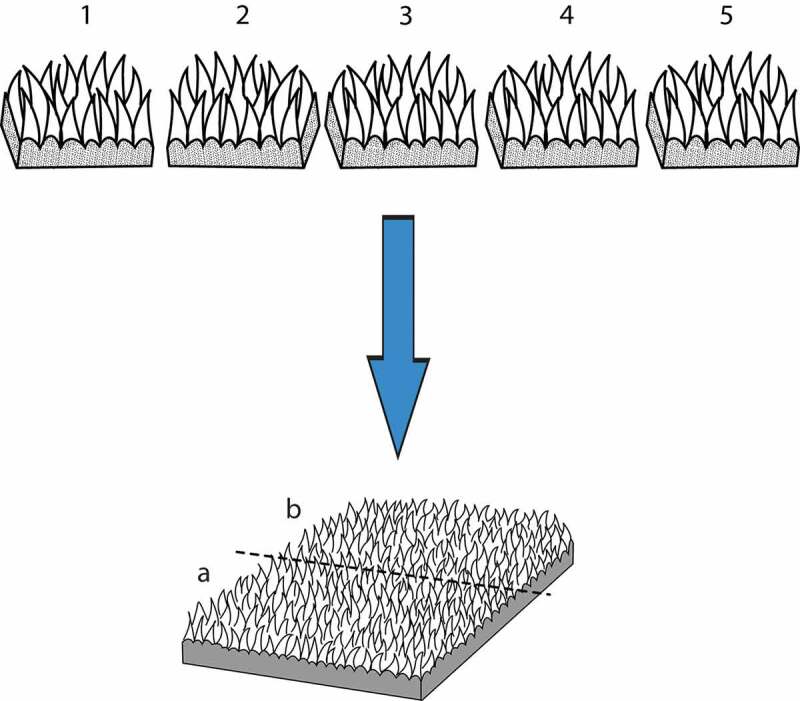
Schema showing systematic sampling of the ruminal tissue. A slice is selected randomly (3 in this case) from the interval 1–5 then every 5^th^ slice is picked until the entire ruminal sac is exhaustively sampled. Each of the slices is cut into two roughly equal halves (dashed line) and one of the slices, (for example “A”), is used for vertical sections while the other one (b) is used for horizontal sections. The slices were rotated about a vertical axis before embedding to achieve true horizontal and vertical uniform random orientation. This was achieved by placing the slice in a petri dish and spinning the petri dish

The ruminal sac was placed with its serosal surface on a dissection wax plate while the mucosal surface faced up. It was serially cut transversely to obtain long slices of tissue at intervals of approximately 5 cm apart. The transverse slices were serially cut in a longitudinal direction (i.e. perpendicular to the first cut) at intervals of 3 cm to ultimately obtain smaller rectangular slices of the dorsal sac (Figure 3). From the total number of the rectangular blocks of tissue obtained, a sub-sample was selected through systematic random sampling [12]. The first block of tissue to be selected among the first five blocks was determined by randomly picking a number between 1 and 5 (Figure 4). Thus the number picked determined the starting position for selecting the first block and subsequently every fifth additional tissue block was selected from the remaining lot. Selecting every fifth block of tissue was done serially through each row in alternate left and right directions for the successive rows. Thus if the first row was sampled from left to the right, the second row was sampled from right to the left; and this was continued alternately for all successive rows until the sampling of the entire rumen sac was accomplished. Each of the remaining rumen sacs was sliced and sampled as described for the dorsal sac. Each tissue slice obtained was divided into two roughly equal parts one of which was processed for vertical sections and the other one for horizontal sections (Figure 4). The five blocks of tissue from each ruminal sac were placed as a group in individually labelled containers with 10% NBF solution and stored until processing time.

### 2.8. Histology

**Figure 5.**
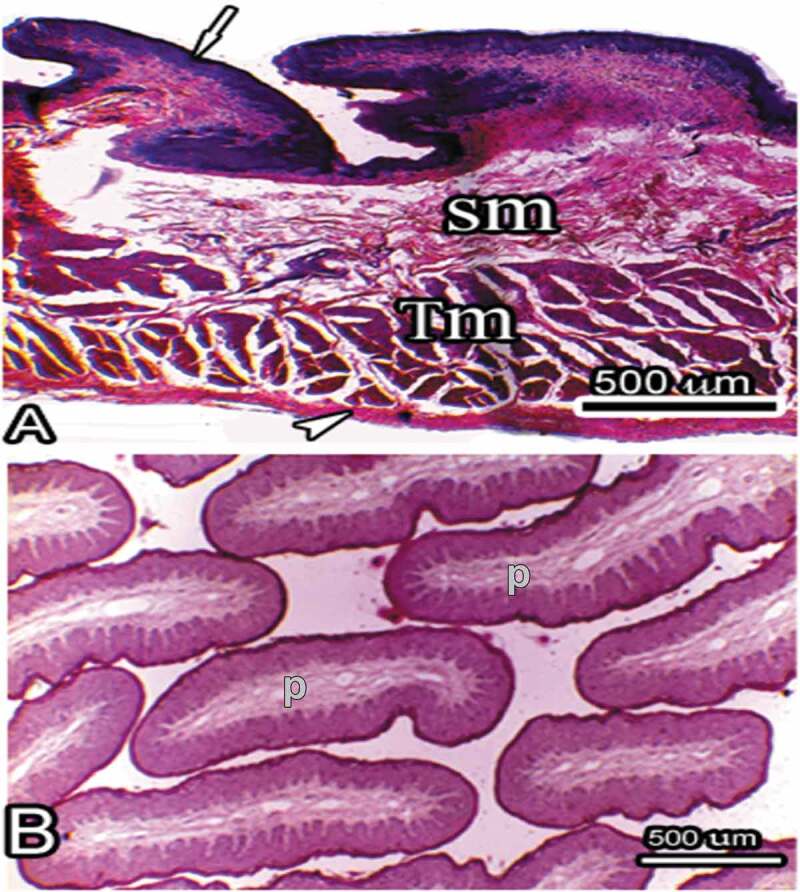
Histological micrographs showing a vertical section (a) and a horizontal section (b). Note that the vertical section captures the entire wall thickness as well as the lengths of the projecting papillae. This section is used for estimation of volume and surface densities. The horizontal section is obtained perpendicular to the long axes of the papillae and as such shows only the profiles of transected papillae (P). It is used for estimation of papillary packing densities. The tissue layers denoted are the mucosa (arrow), the submucosa (Sm), the tunica muscularis (Tm) and the serosa (arrow-head)

The selected tissue blocks were dehydrated through ascending concentrations of ethanol starting with 70% to 100%, then in acetone and cleared in methyl benzoate to allow for infiltration process. The blocks were infiltrated with molten paraffin and then embedded in paraffin wax. Embedding was done with the preferred orientation either to generate vertical sections or horizontal sections (Figure 5). Subsequently, sections were obtained at a nominal thickness of 5 um and then stained with haematoxylin and eosin (H/E) for observation under the light microscope. Digital images were obtained with a Leica ICC-50 Digital Light Microscope using Leica **LAS EZ** software.

#### 2.8.1. Volume density and volume estimation

**Figure 6.**
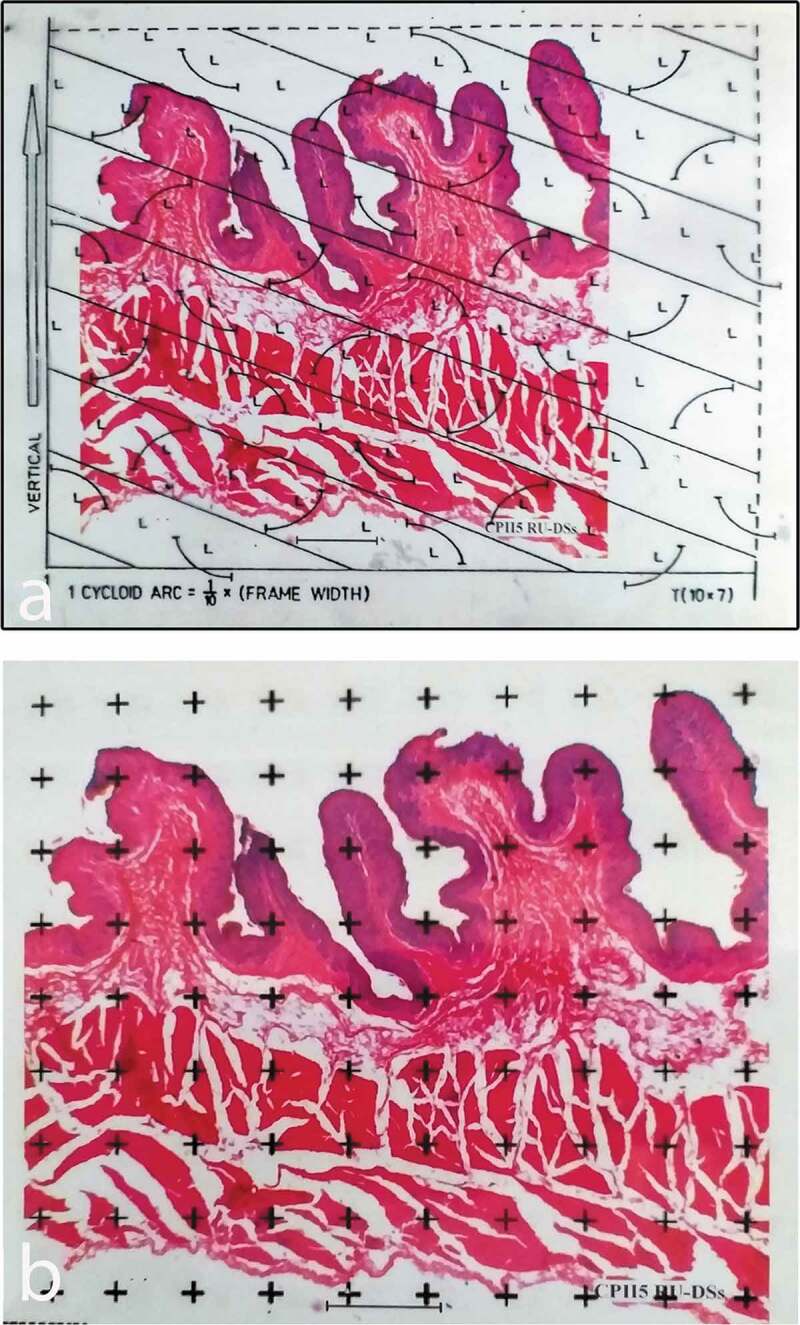
Histological micrographs showing a vertical section (a) with superimposed cycloid arc lattice grids for surface density (Sv) estimation and with a superimposed set of points (b) for estimating volume densities. The points are electronically generated using the STEPanizer software (Tschanz *et al*. 2011)

Digital micrographs of ruminal sac vertical sections were used to estimate volume densities of the tunica mucosa, the tunica submucosa, the tunica muscularis and the tunica serosa with a point-counting stereological grid (Figure 6). The surface density of the ruminal mucosa was estimated using a cycloid test system. The stereological estimates were obtained using STEPanizer software and the procedure for counting individual parameters were followed as described in the flow chart by Tschanz et al. [9]. The volume density Vv, of a component was determined by dividing the number of points falling on the particular layer in the rumen wall by the number of points falling on the entire rumen wall [13] and expressed as a percentage.

Volume density was calculated as follows,

$$Vv(Co,Ref)=\frac{\sum Pt(Co)}{\sum Pt(Ref)}*100$$

where,

Vv(Co,Ref) is the volume density of the tissue component of the wall of the ruminal sac,

∑Pt(Co) is the total number of points falling on the profiles of the tissue component,

∑Pt(Ref) is the total number of points falling on the entire rumen wall.

Absolute volume of a tissue component in the wall of the ruminal sac was estimated as follows,

VCo=VvCo∗Vref

Where,

VCo is the absolute volume of the tissue component (e.g. mucosa) in the ruminal sac;

VvCo is the volume density of tissue component (e.g. mucosa) in the wall of ruminal sac;

Vref is reference volume of the ruminal sac.

For example,

V(mucosa)=Vv(mucosa)∗V(sac)

##### 2.8.1.1. Absolute volume of a tissue component in an entire rumen per animal

This was calculated as follows,

$$V_{\left(Muc,Tot\right)}=V_{\left(muc,AR\right)}+V(muc,DS)\text{\textbackslash{}break}{\;}_{\;}+V(muc,VS){\;}_{\;}+V(muc,CDB){\;}_{\;}+V(muc,CVB){\;}_{\;}$$

Where,

$V(muc,Tot){\;}_{\;}$ is the total absolute volume of tissue (e.g. mucosa) in the rumen;

$V_{\left(muc,AR\right)}$is the absolute volume of tissue in the cranial sac;

$V(muc,DS){\;}_{\;}$is the absolute volume of tissue in the dorsal sac;

$V(muc,VS){\;}_{\;}$ is the absolute volume of tissue in the ventral sac;

$V(muc,CDB){\;}_{\;}$ is the absolute volume of tissue in the caudo-dorsal blind sac;

$V(muc,CVB{)}_{\;}i$s the absolute volume of tissue in the caudo-ventral blind sac.

#### 2.8.2. Surface density and surface area estimation

For surface density estimates, a cycloid test system was superimposed on the projected image of ruminal sac (Figure 6) with the vertical axis of the tissue and that of the test system running parallel [14]. The vertical axis of the tissue was identified as the general direction parallel to the long axis of the ruminal papillae. The number of intersections of the cycloid arcs with the boundary of the mucosal surface of the ruminal sac was counted.

Surface density of each ruminal sac was calculated as follows;

$$Sv=2*\frac{\sum i}{l/p*\sum P}$$

where,

Sv is the surface density of the ruminal sac;

∑i is the total number of intersections between the cycloid test lines and the mucosal surface for the ruminal sac;

l/p is the test line length per point.

∑P is the total number of points hitting the ruminal surface.

Absolute surface area of each ruminal sac was calculated as follows,

SAsac=Sv∗Vref

where,

SAsac is the absolute surface area of the ruminal sac;

Sv is the surface density of the ruminal sac;

Vref is the reference volume of the ruminal sac.

Total surface area of the entire rumen per animal was calculated as follows,

$$S_{Tot}=S_{AR}+S_{DS}+S_{VS}+S_{CDB}+S_{CVB}$$

where,

$S_{Tot}$ is the total surface area of the entire rumen per animal;

$S_{AR}$ is the surface area of cranial sac;

$S_{DS}$ is the surface area of dorsal sac;

$S_{VS}$ the surface area of ventral sac;

$S_{CDB}$ is the surface area of caudodorsal blind sac;

$S_{CVB}$ is the surface area of caudoventral blind sac.

#### 2.8.3. Numerical density of papillae and papillae number estimation

**Figure 7.**
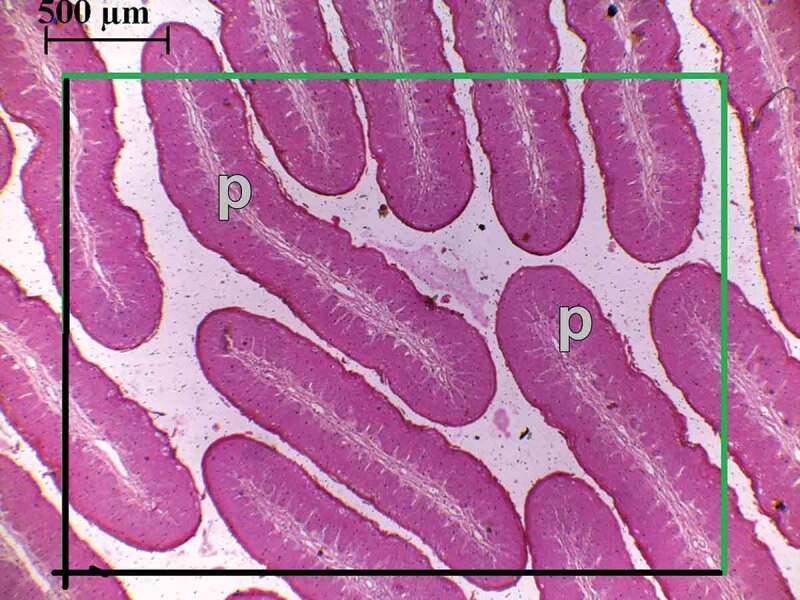
A histological micrograph showing a horizontal section with a superimposed counting frame and papillary profiles (P). All profiles within the frame together with those touching the green line are counted but not those touching the black line (forbidden line)

For the packing (numerical) density estimates, a counting frame with a forbidden line was superimposed on the projected image of horizontal sections [15]. All the profiles within the frame and all those touching the green line but not the black line were counted (Figure 7). The area of the counting frame in real units was estimated and the numerical density calculated. Sampling at the image level was done in a systematic way such that counting windows were generated starting from the top left corner of the image, moving in the right side while keeping a constant step.

Numerical density of papillae per counting frame was calculated as follows,

$$N_{A}=\frac{Q-}{A}$$

Where,

$N_{A}$ is the numerical density of the ruminal papillae per sac;

Q- is the total number of countable papillae profiles per counting frame and

A is the area of the counting frame in real units.

Where there is a different magnification, the equation changes to,

$${\text{N}}_{\text{A}}\text{=}\frac{\text{Q}-}{\text{A}}*1/\text{M}$$

To find the average packing density of papillae per sac, the total number of profiles counted was divided by the total area of the counting frames.

Thus;

Total number of papillae for each ruminal sac was calculated as follows,

$${\text{N}}_{\text{A}}(\text{pap})\text{=}\frac{\sum Q-}{\sum \text{A}}*1/\text{M}$$

N(pap) is the total number of the ruminal papillae per sac;

∑Q- is the total number of countable papillae profiles per sac

∑A is the area of the counting frames

M is magnification

Alternatively, the total number of papillae per sac can be expressed as follows:

N(pap,Sac)=NA∗Smsac

Where,

N(pap,sac) is the number of papillae in the ruminal sac;

N_A_ is the numerical density of papillae in the sac;

Smsac is the primary surface area of the ruminal sac.

Total number of papillae for the entire rumen per animal was calculated as follows,

$$N_{Tot}=N_{AR}+N_{DS}+N_{VS}+N_{CDB}+N_{CVB}$$

Where,

$N_{Tot}$ is the total number of papillae in the entire rumen per animal;

$N_{AR}$ is the total number of papillae in the cranial sac;

$N_{DS}$ is the total number of papillae in the dorsal sac;

$N_{VS}$ is the total number of papillae in the ventral sac;

$\;N_{CDB}$ is the total number of papillae in the caudodorsal blind sac;

$N_{CVB}$ is the total number of papillae in the caudoventral blind sac.

#### 2.8.4. Papillary amplification factors

The papillary amplification factor is an indicator of how the papillae increase the primary surface area of the rumen. This parameter was estimated retrospectively by dividing the absolute surface area with the primary surface area of the rumen.

In previous studies amplification factors were estimated as a ratio of intersections between the amplifying structures to those with the primary surface [15–17].

The amplification factor AF is defined as,

$$\frac{\text{AF}[\text{pap}]\text{=SAsac}}{\text{Sm}}$$

Where,

SAsac is the absolute surface area of the ruminal sac;

Sm is the primary surface area of the ruminal sac.

### 2.9. Statistical analysis

To test the robustness of these methods, both the coefficient of variation (CV) and the coefficient of error (CE) were calculated for each parameter. The coefficient of variation represents the ratio of the standard deviation to the mean, and it is a useful statistic for comparing the degree of variation from one data series to another, even if the means are drastically different from one another. The lower the CV, the better the estimate. When the mean value is close to zero, the coefficient of variation will approach infinity and is therefore sensitive to small changes in the mean. However, the CV does not depend on the sample size [8]. Conversely, the CE is a measure of how good the estimate is and it is a standard statistical value that is used extensively in stereology. It is defined as the standard error of the mean of repeated estimates divided by the mean.

Thus;

CE = SE/µ

Where

SE is standard error of mean

µ is mean of the sample

## 3. Results

The morphometric results in this study show the efficiency of the techniques described and their applicability and important trends in some of the parameters and for the first time document some basic stereological parameters of the ovine rumen under normal feeding regiment. In most cases, the coefficient of variation remained well below 10% as did the coefficient of error, showing that the estimates were quite robust.

The body weights of the five animals used in the study did not show significant changes during the acclimatization period. The mean body weight of the sheep after 5 weeks of acclimatization was 26.7 ± 2.0 Kg.

### 3.1. Volumes of ruminal sac tissues, macroscopic surface areas, total number of papillae and absolute surface areas

The mean volume of ruminal tissue was 536.54 ± 80.52 cm^3^. The mean ruminal macroscopic surface area was 1091.25 ± 103.53 cm^2^. Total number of papillae was 92,884.91 ± 6216.46, and the mean absolute surface area was 4726.74 ± 628.56 cm^2^. The details are provided in Table 1 .

**Table 1.** Mean volumes, macroscopic and absolute surface areas as well as papillary numbers in the various ruminal sacs. Total values for the entire rumen are also provided. In this and subsequent tables, the standard deviations are given in parentheses

	Ruminal sac					
Parameter	AR	DS	VS	CDB	CVB	Total
Tissue volumes (cm^3^)	112.35 (13.37)	111.88 (20.3)	201.34 (30)	19.75 (3.07)	91.22 (19.15)	536.54 (80.52)
Macroscopic surface areas (cm^2^)	220.5 (31.74)	277.65 (19.39)	334.8 (46.53)	48.15 (4.93)	210.15 (40.05)	1091.25 (103.53)
Papillary numbers	20,402.62 (3475.76)	23,484.86 (4431.03)	26,237.78 (3699.73)	3565.42 (635.84)	19,194.24 (4186.76)	92,884.91 (6216.46)
Absolute surface areas (cm^2^)	1012.53 (214.14)	900.67 (146.76)	1864.18 (233.42)	160.23 (21.72)	789.13 (146.4)	4726.74 (628.56)

### 3.2. Mucosal surface densities, papillary packing densities and papillary amplification factors

The surface density of the mucosa was 8.68 ± 0.89 cm^−1^. The mean papillary packing densities did not appear to have a trend and ranged from 73.67 ± 9.02 in the CDB to 92.54 ± 11.65 cm^−2^ in the AR. The mean papillary packing density for the entire rumen was 84.64 ± 10.99 cm^−2.^ The extent to which the papillae increased the primary surface area was estimated retrospectively by dividing the absolute surface area of the mucosa by the macroscopic surface area of the respective ruminal sac. Mean amplification factors were highest in the ventral sac at 5.68 ± 1.11 and lowest in the dorsal sac at 3.23 ± 0.32 (Table 2).

**Table 2.** Mucosal surface densities, papillary packing densities and papillary amplification factors in the various ruminal sacs. Values for the entire rumen are also provided. All values are given as mean ± SD

	Ruminal sac					
Parameter	AR	DS	VS	CDB	CVB	MEAN
Mucosal surface density (cm^−1^)	9.0 (1.47)	8.10 (0.66)	9.40 (1.66)	8.18 (1.03)	8.74 (1.19)	8.68 (0.89)
Papillary packing densities (cm^−2^)	92.54 (11.65)	84.88 (19.12)	79.72 (16.32)	73.67 9.02	92.35 (20.89)	84.64 (10.99)
Papillary amplification factors	4.58 (0.59)	3.23 (0.32)	5.68 (1.11)	3.36 (0.48)	3.79 (0.48)	4.13 (0.47)

### 3.3. Volume densities of the tissue layers of the ruminal walls

The volume densities of the tissues constituting the ruminal wall show that the tunica muscularis takes the largest proportion at about 47.5%, followed by the mucosa at 29.6%, then the submucosa at 20.3% and the serosa is the smallest at 2.6%. The details are provided in Table 3 .

**Table 3.** Volume densities (Mean ± SD) of the mucosa, submucosa, tunica muscularis and the serosa. Mean values for the entire rumen are also provided

	Ruminal sac					
Volume density (%)	AR	DS	VS	CDB	CVB	MEAN
Mucosa	29.58 (4.95)	30.02 (4.45)	28.38 (5.02)	31.24 (5.6)	32.24 (4.29)	29.58 (3.54)
Submucosa	16.64 (2.17)	19.68 (1.27)	20.36 (4.38)	21.96 (3.02)	22.64 (1.24)	20.26 (1.03)
Muscularis	50.72 (5.26)	47.66 (4.26)	49.38 (6.42)	43.98 (5.07)	42.58 (4.17)	47.5 (3.62)
Serosa	3.02 (0.46)	2.64 (0.70)	1.88 (0.82)	2.8 (0.72)	2.54 (0.69)	2.58 (0.35)

### 3.4. The volumes of the tissue layers constituting the ruminal wall

The volumes of the tissue layers constituting the ruminal wall in the various sacs as well as the ruminal totals are provided in Table 4. The tunica muscularis was consistently the largest component at 256.17 ± 23.3 cm^3^ followed by the mucosa at 162.49 ± 45.07 cm^3^, then the submucosa at 106.67 ± 17.3 cm^3^ and the least was the serosa at 13.21 ± 3.4 cm^3^.

**Table 4.** Volumes (Mean ± SD) of the layers of the ruminal wall provided for the various sacs. Mean values for the entire rumen are also included

	Ruminal sac					
Volume (cm^3^)	AR	DS	VS	CDB	CVB	TOTAL
Mucosa	33.57 (8.64)	34.17 (10.42)	57.97 (18)	6.93 (2.45)	29.85 (9.54)	162.49 (45.07)
Submucosa	18.66 (2.96)	22.1 (4.68)	40.58 (9.08)	4.79 (1.26)	20.53 (3.75)	106.67 (17.3)
Muscularis	56.71 (5.97)	52.7 (5.78)	98.87 (15.96)	9.41 (1.06)	38.49 (6.68)	256.17 (23.3)
Serosa	3.414 (0.66)	2.912 (0.67)	3.91 (1.94)	0.62 (0.27)	2.35 (0.94)	13.21 (3.4)

## 4. Discussion

The data obtained in this study have established basic rumen parameters and demonstrated what is achievable with the methods described. Many previous morphometric studies have used model-based techniques to quantify ruminal tissue [18,19]. As reported elsewhere, many model-based designs are laden with systematic errors [8] and indeed many such studies were based on small portions or components of the rumen [6,20].

The present study provides a comprehensive set of methods and approaches for quantifying ruminal structure at macroscopic and histological levels. The STEPanizer software [9], which is freely available online was used to stereologically analyse histological sections. The reference volume is easily obtained using the weight displacement method [11] or even the Cavalieri method. In the current case, reference volumes for individual sacs were determined using the former method.

The macroscopic surface areas on the other hand were estimated using the point associated area method [8]. Vertical sections and cycloid arcs are a convenient combination for estimating surface area [14]. The cycloid arcs were placed electronically using the STEPanizer software and the surface density determined from intersection counts as detailed above. The absolute surface area was determined by multiplying the surface density with the reference volume.

The numerical density of papillae (packing density) was estimated as a two-dimensional parameter. Since papillae are discrete anisotropic structures, generation of horizontal uniform random sections perpendicular to the long axes of the papillae captures all the profiles of the papillae, provided that the sections are obtained at mid-length of the papillae. Previously we have demonstrated estimation of number for intestinal microvilli from their transected profiles [15]. In all cases, 2D counting frames and the forbidden line rule [21] are applied. Systematic random sampling is done within the tissue sections using counting frames and between 100 and 200 papillary profiles per individual are counted. To estimate the total number of papillae per sac, the papillary packing density, N_A_ (pap), is multiplied by the macroscopic surface area of the sac.

An additional parameter estimated here was the papillary amplification factor, an indicator of how the papillae increase the primary surface area of the rumen. This parameter was estimated by dividing the absolute surface area with the primary (mucosal) surface area of the rumen. It can be used as an indicator of how healthy the mucosa is. Previously, amplification factors were estimated for intestinal villi [22] and microvilli [16,17,23].

Several studies have shown that papillary morphometry can vary with the type of diet [24–26] especially in growing animals. It is therefore imperative that studies specify the type of feed used and that such feeding regimens be maintained in experimental groups. It is, however, expected in normal standard feeding regimens, data on ruminal morphometry should be reproducible.

Both the coefficient of variation (CV) and the coefficient of error (CE) were calculated for each parameter in this study to test the robustness of these methods. Generally, the lower the CV, the better the estimate. Many parameters, and for most of the sacs analysed, CV remained, well below 20%, showing that the dispersion remained remarkably low [27,28].

The relationship between the observed variation (OCV) is as a result of biological variation (BCV) and sampling variation coefficient of error (CE) [8,29].

Thus;

OCV^2^ ≅ BCV^2^ + CE^2^

Increasing the number of animals per sample has no effect on BCV but affects the sampling variation, CE. In most cases in this study, the CE remained well below 10% (data not shown).

Paraffin embedding is known to cause substantial tissue shrinkage. Tissue shrinkage may be estimated from the formula described by Nyengaard [30] and is the volume change observed after embedding. However, for the coarse parameters estimated in the current study, tissue shrinkage may be of little consequence since reference volumes and surfaces were estimated before embedding, provided that all tissue layers have the same degree of shrinkage on paraffin infiltration. To avoid problems of tissue shrinkage, firm resins such as glycol methacrylate are recommended [31]. Elsewhere, tissue osmication and prolonged staining with uranyl acetate prior to dehydration and embedding in plastic have been found to greatly diminish shrinkage in ultrastructural studies [32].

## 5. Conclusion

This study shows interesting trends in the morphometrics of the rumen structure. When ruminal sacs were compared in terms of volumes, macroscopic and absolute surface areas, ranking appears to be in the order VS>DS>AR>CVB>CDB. Papillary packing densities did not appear to have any trend, but total numbers were highest in the VS and lowest in the CDB. The methods documented here are unbiased and devoid of assumptions and it is now possible to do a detailed stereological analysis of ruminal tissue in different experimental or even pathological conditions.

## References

[1] Bergman EN. Energy contributions of volatile fatty acids from the gastrointestinal tract in various species. Physiol Rev. 1990;70(2):567–590. 2181501 10.1152/physrev.1990.70.2.567

[2] Lentle RG The use of anatomical features of the stomach to investigate the nutritional status of deer populations, MSc. Thesis, Massey University, New Zealand, 1994.

[3] Poonia A, Kumar P, Kumar P Histomorphological studies on the rumen of the sheep, *Ovis Aries*. Haryana Vet. 2011;50:49–52.

[4] Singh N, Puri JP, Nangia OP, et al Early development of rumen function in buffalo calves. 4. Rumen microbes, metabolism and cellulose digestion in vitro as a function of age and diet. Indian J Anim Sci. 1983;53:933–936.

[5] Steven DH, Marshall AB Organization of the rumen epithelium In: Phillipson AT, editor. Physiology of digestion and metabolism in the ruminant. Newcastle Upon Tyne, England: Oriel Press; 1970 p. 80–100.

[6] Scott A, Gardner IC Papillar form in the forestomach of the sheep. J Anat. 1973;116(Pt 2):255–267. 4783418 PMC1271600

[7] Yamamoto Y, Atoji Y, Agungpriyono S, et al Morphological study of the forestomach of the Japanese serow (*Capricornis crispus*). Anat Histol Embryol. 1998;27(2):73–81. 9591368 10.1111/j.1439-0264.1998.tb00160.x

[8] Howard CV, Reed MG Unbiased stereology, three-dimensional measurement in microscopy. New York: Advanced Methods Garland Science/BIOS publishers; 2005.

[9] Tschanz SA, Burri PH, Weibel ER A simple tool for stereological assessment of digital images: the STEPanizer. J Microsc. 2011;243(1):47–59. 21375529 10.1111/j.1365-2818.2010.03481.x

[10] McGavin MD, Morrill JL Dissection technique for examination of the bovine 10. ruminoreticulum. J Anim Sci. 1976;42(2):535–538. 1262269 10.2527/jas1976.422535x

[11] Scherle W A simple method for volumetry of organs in quantitative stereology. Mikroskopie. 1970;26(1):57–60. 5530651

[12] Cruz-Orive LM, Weibel ER Sampling designs for stereology. J Microsc. 1981;122(Pt 3):235–257. 7017151 10.1111/j.1365-2818.1981.tb01265.x

[13] Gundersen HJ, Boysen M, Reith A Comparison of semiautomatic digitizer-tablet and simple point counting performance in morphometry. Virchows Arch B Cell Pathol Incl Mol Pathol. 1981;37(3):317–325. 6117976 10.1007/BF02892580

[14] Baddeley AJ, Gundersen HJ, Cruz-Orive LM Estimation of surface area from vertical sections. J Microsc. 1986;142(Pt 3):259–276. 3735415 10.1111/j.1365-2818.1986.tb04282.x

[15] Makanya AN, Self TJ, Warui CN, et al Gut morphology and morphometry in the epauletted Wahlberg’s fruit bat (*Epomophorus wahlbergi*, Sundevall, 1846). Acta Biol Hung. 2001;52(1):75–89. 11396843 10.1556/ABiol.52.2001.1.8

[16] Makanya AN, Mayhew TM, Maina JN Stereological methods for estimating the functional surfaces of the chiropteran small intestine. J Anat. 1995;187(Pt 2):361–368. 7591999 PMC1167431

[17] Makanya AN, Maina JN, Mayhew TM, et al A stereological comparison of villous and microvillous surfaces in small intestines of frugivorous and entomophagous bats: species, inter-individual and craniocaudal differences. J Exp Biol. 1997;200(Pt 18):2415–2423. 9343854 10.1242/jeb.200.18.2415

[18] Steele MA, Garcia F, Lowerison M, et al Technical note: three-dimensional imaging of rumen tissue for morphometric analysis using micro-computed tomography. J Dairy Sci. 2014;97(12):7691–7696. 25262181 10.3168/jds.2014-8374

[19] Melo LQ, Costa SF, Lopes F, et al Rumen morphometrics and the effect of digesta pH and volume on volatile fatty acid absorption. J Anim Sci. 2013;91(4):1775–1783. 23345561 10.2527/jas.2011-4999

[20] Shen Z, Seyfert HM, Löhrke B, et al An energy-rich diet causes rumen papillae proliferation associated with more IGF type 1 receptors and increased plasma IGF-1 concentrations in young goats. J Nutr. 2004;134(1):11–17. 14704286 10.1093/jn/134.1.11

[21] Gundersen HJ The new stereological tools. Acta Pathol Microbiol et Immunol Scand. 1988;96: 857–881. 10.1111/j.1699-0463.1988.tb00954.x 3056461

[22] Mayhew TM Geometric model of the rat intestinal mucosa for stereological evaluation of villus amplification factors. J Microsc. 1984;135(Pt 3):337–346. 6492144 10.1111/j.1365-2818.1984.tb02538.x

[23] Mayhew TM Quantitative ultrastructural study on the responses of microvilli along the small bowel to fasting. J Anat. 1987;154:237–243. 3446662 PMC1261849

[24] Scocco P, Ceccarelli P, Gatti R, et al Use of a geographic information system to evaluate morphometric variations of rumen papillae related to diet and pasture vegetative cycle. Vet Ital. 2007;43(3):425–429. 20422518

[25] Scocco P, Mercati F, Tardella FM, et al Increase of forage dryness induces differentiated anatomical response in the sheep rumen compartments. Microsc Res Tech. 2016;79(8):738–743. 27271434 10.1002/jemt.22692

[26] Lima TJ, Costa RG, de Medeiros GR, et al Ruminal and morphometric parameters of the rumen and intestines of sheep fed with increasing levels of spineless cactus (*Nopalea cochenillifera* Salm-Dyck). Trop Anim Health Prod. 2019;51(2):363–368. 30168022 10.1007/s11250-018-1697-1

[27] Nyengaard JR, Alwasel SH Practical stereology of the stomach and intestine. Ann Anat. 2014;196(1):41–47. 24365710 10.1016/j.aanat.2013.10.007

[28] Cui ZC Allowable limit of error in clinical chemistry quality control. Clin Chem. 1989;35(4):630–631. 2702747

[29] Santos M, Marcos R, Santos N, et al An unbiased stereological study on subpopulations of rat liver macrophages and on their numerical relation with the hepatocytes and stellate cells. J Anat. 2009;214(5):744–751. 19438768 10.1111/j.1469-7580.2009.01055.x PMC2707097

[30] Nyengaard JR Stereologic methods and their application in kidney research. J Am Soc Nephrol. 1999;10(5):1100–1123. 10232698 10.1681/ASN.V1051100

[31] Gerrits PO, Horobin RW Glycol methacrylate embedding for light microscopy, basic principles and trouble-shooting. J Histotechnol. 1996;19(4):297–311.

[32] Ochs M A brief update on lung stereology. J Microsc. 2006;222(Pt 3):188–200. 16872418 10.1111/j.1365-2818.2006.01587.x

